# A violation of universality in anomalous Fourier’s law

**DOI:** 10.1038/srep38823

**Published:** 2016-12-13

**Authors:** Pablo I. Hurtado, Pedro L. Garrido

**Affiliations:** 1 Institute Carlos I for Theoretical and Computational Physics and Departamento de Electromagnetismo y Física de la Materia, Universidad de Granada, 18071 Granada, Spain

## Abstract

Since the discovery of long-time tails, it has been clear that Fourier’s law in low dimensions is typically anomalous, with a size-dependent heat conductivity, though the nature of the anomaly remains puzzling. The conventional wisdom, supported by renormalization-group arguments and mode-coupling approximations within fluctuating hydrodynamics, is that the anomaly is universal in 1*d* momentum-conserving systems and belongs in the Lévy/Kardar-Parisi-Zhang universality class. Here we challenge this picture by using a novel scaling method to show unambiguously that universality breaks down in the paradigmatic 1*d* diatomic hard-point fluid. Hydrodynamic profiles for a broad set of gradients, densities and sizes all collapse onto an universal master curve, showing that (anomalous) Fourier’s law holds even deep into the nonlinear regime. This allows to solve the macroscopic transport problem for this model, a solution which compares flawlessly with data and, interestingly, implies the existence of a bound on the heat current in terms of pressure. These results question the renormalization-group and mode-coupling universality predictions for anomalous Fourier’s law in 1*d*, offering a new perspective on transport in low dimensions.

It’s going to be 200 years since Fourier stated his seminal law^1^, but its microscopic understanding still poses one of the most important and challenging open problems in nonequilibrium statistical physics, with no rigorous mathematical derivation to date^2^,^3^,^4^,^5^,^6^,^7^. Fourier’s law establishes the proportionality between the heat current and the local temperature gradient in a material, with the proportionality factor defining the heat conductivity *κ*, a key material property. While for bulk, three-dimensional materials *κ* is well characterized and measured, its status in low-dimensional structures is far from clear. In particular, for low-dimensional systems (*d* = 1, 2) with momentum conservation, the effective conductivity *κ* grows with the system size *L*, diverging in the thermodynamic limit and thus leading to anomalous heat transport^3^,^4^,^5^,^6^,^7^. The understanding of this anomaly has attracted a lot of attention in recent years^3^,^4^,^5^,^6^,^7^,^8^,^9^,^10^,^11^,^12^,^13^,^14^,^15^,^16^,^17^,^18^,^19^,^20^,^21^,^22^,^23^,^24^,^25^,^26^,^27^,^28^,^29^,^30^,^31^,^32^,^33^,^34^,^35^,^36^,^37^,^38^,^39^,^40^,^41^, not only because it is expected to shed light on the key ingredients behind Fourier’s law at a fundamental level, but also because of its technological relevance in low-dimensional real-world materials, the most noteworthy being graphene^8^,^9^,^10^,^11^,^12^, but with other important examples ranging from molecular chains^13^ and carbon nanotubes^14^ to polymer fibers^15^,^16^, nanowires^17^,^18^ and even spider silk^19^, to mention just a few; see^7^ for a recent review. From a theoretical perspective, the low-dimensional anomaly in heat transport can be linked to the presence of strong dynamic correlations in these fluids and lattices^20^,^21^,^22^, though a detailed understanding has remained elusive for decades.

In 1*d*, clear signatures of this anomaly appear in a number of different phenomena^42^. For instance, the steady state heat current *J* of a 1*d* momentum-conserving system driven by a *small* boundary temperature gradient (i.e. in the linear regime) typically scales as *L*^−1+*γ*^ for large enough system sizes *L*, with 0 ≤ *γ* < 1 an anomaly exponent, which can be interpreted in terms of a finite-size heat conductivity *κ*_*L*_ ~ *L*^*γ*^. An exponent *γ* = 0 corresponds to standard diffusive transport, but typically *γ* > 0 is observed in 1*d* implying superdiffusive heat transport^42^. The low-dimensional transport anomaly is also apparent in equilibrium. In particular, the long-time tail of the equilibrium time correlation of the energy current decays in 1*d* in a nonintegrable, power-law way, 〈*J*(0)*J*(*t*)〉 ~ *t*^−1+*δ*^ as *t* → ∞, with 0 ≤ *δ* < 1 another exponent. Green-Kubo relations for the transport coefficients hence imply a divergent value for the heat conductivity, in agreement with nonequilibrium results^6^. Additional signatures of anomalous transport have been also reported in the superdiffusive spreading of energy perturbations in equilibrium^40^,^41^,^42^,^43^. A range of different values for the exponents *γ* and *δ* have been measured in simulations and experiments for different model systems^4^,^5^,^6^,^7^, the main difficulty being extracting the large *L* asymptotics due to the strong and poorly understood finite-size effects affecting these measurements (which mix bulk and boundary finite-size corrections). The prevailing picture, however, is that the transport anomaly exponents are *universal* and within the Lévy/Kardar-Parisi-Zhang (L/KPZ) universality class^3^,^4^,^5^,^6^,^7^, a conjecture based on renormalization-group^23^ and mode-coupling^24^ calculations, and reinforced by recent related breakthroughs from nonlinear fluctuating hydrodynamics^25^,^26^,^27^,^28^,^29^ which predict Lévy (KPZ) scaling for the heat (sound) modes of the equilibrium time correlators of *conserved fields*. In particular, for the transport anomaly *γ* = 1/3 = *δ* is expected in the general case, though a second universality class with *γ* = 1/2 = *δ* seems to appear under special circumstances (as e.g. for zero-pressure systems with symmetric potential^24^,^30^,^31^,^32^,^33^,^34^). Special cases with convergent *κ* in 1*d*, as the coupled rotors model^44^,^45^, can be also accounted for by fluctuating hydrodynamics after noticing that these models have less than three locally-conserved fields^46^.

In this work we challenge the universality conjecture for anomalous Fourier’s law by using a novel scaling method to offer a high-precision measurement of the conductivity anomaly in a paradigmatic 1*d* model of transport. Compared to previous attempts at characterizing the transport anomaly, most based on *linear* response theory and hence critically-dependent on a large system-size limit (which is in fact never attained)^39^, our method takes full advantage of the *nonlinear* character of the heat conduction problem in a natural way, allowing to disentangle the crucial bulk size scaling from the artificial boundary finite-size corrections. Our model is the archetypical 1*d* diatomic hard-point gas in a temperature gradient^47^,^48^,^49^,^50^,^51^,^52^,^53^,^54^,^55^,^56^,^57^, which is characterized by the mass ratio *μ* = *M*/*m* > 1 between neighboring particles. We unambiguously show below that, contrary to the standard lore, this model does obey an anomalous version of Fourier’s law, namely


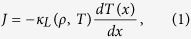


for a broad range of temperature gradients (from the linear response domain to the deeply nonlinear regime), with the heat current *J* proportional to the local temperature gradient via a conductivity functional


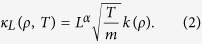


Note that Eqs (1)–(2), 2 are not Fourier’s law in the usual sense, as the latter implies a size-independent *κ*, while the conductivity in this case grows with the system size as *L*^*α*^, with *α* a new exponent characterizing anomalous transport in 1*d*. The validity of Eqs (1)–(2), 2 is proven below by collapsing onto a striking universal master curve the density and temperature profiles measured for a large set of system sizes, number densities and temperature gradients. Such compelling collapse offers a high-precision measurement of the anomaly exponent *α*, which remarkably turns out to be *non-universal*, depending non-monotonously on the mass ratio *μ*. The observed scaling allows to solve the macroscopic transport problem for this model, and we obtain analytic expressions for the universal master curve (as well as for the hydrodynamic profiles, current, pressure, etc.) which exhibit an excellent agreement with measurements. Interestingly, this solution immediately implies the existence of a nontrivial bound on the current in terms of pressure *P*.

A natural question concerns the relation of the new anomaly exponent *α* with the standard ones defined in literature, namely *γ* in the linear response regime and *δ* from equilibrium current time correlations (see description above). This relation can be easily established by studying the linear response limit of the anomalous Fourier’s law (1)–(2), a particular regime of the broad range of temperature gradients where these equations hold with high accuracy, as we demonstrate below. Indeed, for small enough boundary temperature difference Δ*T* the local temperature gradient can be written as *dT*/*dx* ≈ −Δ*T*/*L*, and this together with Eqs (1)–(2), 2 leads to *J* ∝ *L*^−1+*α*^, an argument which strongly suggests the conjecture *α* = *γ*(= *δ*). In this way, the surprising but clear-cut dependence of *α* on the mass ratio *μ* reported below hence signals the breakdown of the universality claimed for 1*d* anomalous Fourier’s law. We maintain here however the different notation for the various (but related) anomaly exponents to stress out their distinct definitions.

## Results

We hence consider a 1*d* Hamiltonian model fluid consisting in *N* hard-point particles with alternating masses, *m* = 1 and *M* = *μm* > 1, moving ballistically in a line of length *L* in between elastic collisions with neighboring particles. The fluid is coupled to two stochastic thermal walls at the boundaries, *x* = 0, *L*, which reflect particles upon collision with a velocity modulus randomly drawn from a Maxwellian distribution defined by the wall temperature *T*_0,*L*_^3^,^4^,^5^,^6^. For *T*_0_ ≠ *T*_*L*_, the temperature gradient drives the system to an inhomogeneous nonequilibrium steady state characterized by nonlinear density and temperature profiles, *ρ*(*x*) and *T*(*x*) respectively^3^,^4^,^5^,^6^,^7^. Interestingly, these profiles can be shown to follow from an universal master curve, independent of the driving gradient and the fluid’s density, if and only if (i) Fourier’s law (1)–(2) and (ii) macroscopic local equilibrium (MLE) hold (see Section I of the Supplementary information for a detailed proof), an equivalence which holds for general *d*-dimensional systems^35^. MLE implies that the stationary density and temperature fields are locally coupled via the equilibrium equation of state (EoS)^58^, which for the 1*d* diatomic hard-point fluid simply takes the ideal gas form, *P* = *ρT*. In this way, iff hypotheses (i)-(ii) hold, we expect all density and temperature profiles to scale as


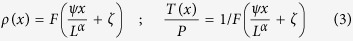


with *ψ* = *J*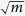/*P*^3/2^ the reduced current and *ζ* a constant, see Section I of the Supplementary information. This scaling defines an universal master curve *F*(*u*) from which all profiles follow. Alternatively, Eq. (3) implies that all measured density and temperature profiles can be collapsed onto an universal master curve after appropriately scaling space by *L*^−*α*^*ψ*, with *ψ* measured in each case, and shifting the curve by a constant *ζ*. The resulting collapse is expected to be very sensitive to the anomaly exponent *α*, and this suggests a simple scaling procedure to measure both *α* and the universal master curve in simulations, confirming at the same time our starting hypotheses.

In order to do so, we performed a large number of event-driven simulations of the 1*d* diatomic gas for a broad set of boundary temperatures *T*_0_ = 2, 5, 10, 20 (with fixed *T*_*L*_ = 1), global number densities *η* ≡ *N*/*L* = 0.5, 1, 2, 3, different mass ratios *μ* = 1.3, 1.618, 2.2, 3, 5, 10, 30, 100, and numbers of particles *N* = 101, 317, 1001, 3163, 10001, reaching up to *N* = 10^5^ + 1 in some cases. We measured locally a number of relevant observables including the local kinetic energy, number density, virial pressure and energy current density, as well as the energy current flowing through the thermal reservoirs at *x* = 0, *L* and the pressure exerted on these walls. We stress that observables measured at the walls agree in all cases with their bulk counterparts, which are constant along the system. For local measurements, we divided the fluid in 30 virtual cells, a constant number independent of other system parameters. The simulation time unit was set to 
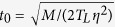
, the mean free time of a heavy particle in a cool environment, and time averages were performed taking into account the relaxation and correlation timescales of the 1*d* fluid, which grow strongly with *N* (see Fig. S4 and Section IIB in the Supplementary information). Statistical errors are computed in all cases at 99.7% confidence level, and error bars are shown if larger than the plotted symbols.

**Figure 1:**
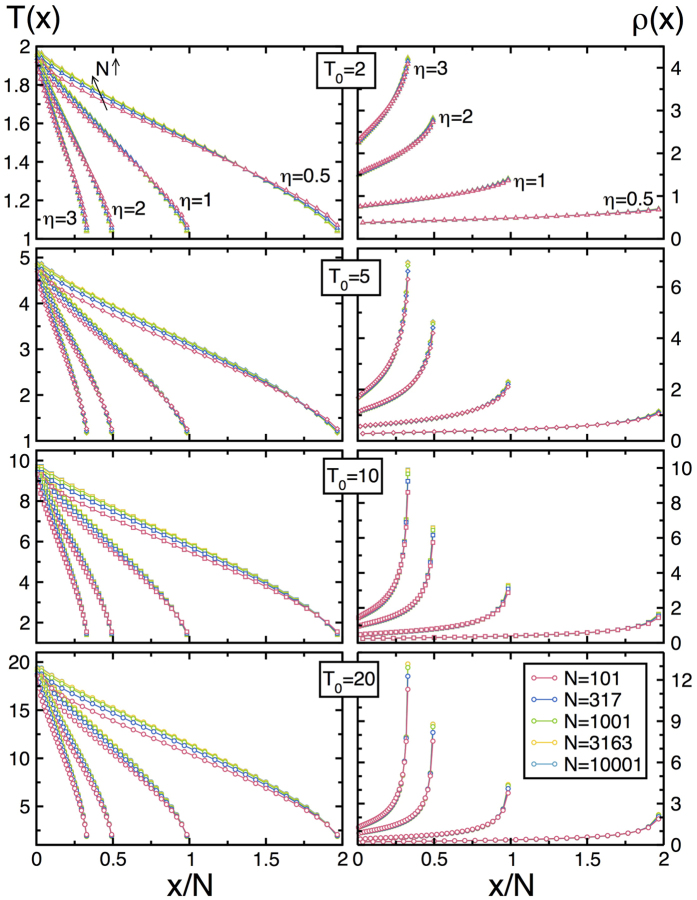
Temperature and density fields under a thermal gradient. Temperature (left) and density (right) profiles measured for (from top to bottom) *T*_0_ = 2, 5, 10, 20 and varying *η* and *N*, for a mass ratio *μ* = 3.

**Figure 2:**
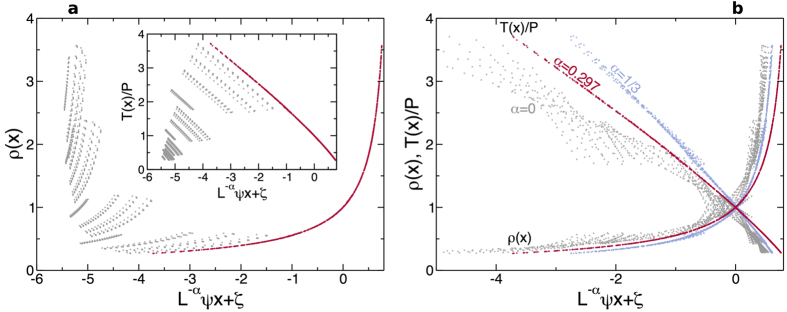
Scaling procedure and data collapse. (**a**) Density profiles for *μ* = 3 ∀ *N, T*_0_, *η* as a function of *L*^−*α*^*ψx* with *α* = 0.297, before (light gray) and after (dark red) the shifts *ζ*. Inset: Same as before, but for the reduced temperature profiles. Note that the shifts are those obtained from density profiles. In both cases the data collapse is remarkable. (**b**) Optimal collapse of density and reduced temperature profiles for *μ* = 3 and three different exponents *α* = 0, 0.297, and 1/3. The superior collapse for *α* = 0.297 is apparent. The abscisa for *α* = 0 has been divided by a factor 10 for the sake of clarity.

**Figure 3:**
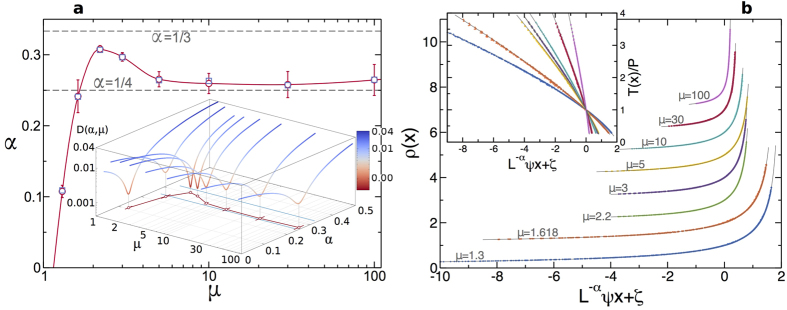
Breakdown of universality and master curves in anomalous Fourier’s law. (**a**) Mass ratio dependence of the anomaly exponent measured from scaling (○). The non-monotonous behavior of *α*(*μ*) clearly signals the breakdown of universality for anomalous Fourier’s law in 1*d*. The exponent measured from the power-law fit for *k*(*ρ*) is also shown (□), being fully compatible with *α* in each case. The line is a guide to the eye. Inset: The collapse metric *D*(*α, μ*) as a function of *α* exhibits a deep and narrow minimum for each *μ* (note the logarithmic scale in *z*-axis), offering a precise measurement of the anomaly exponent and its error. (**b**) Collapse of density profiles for each *μ* obtained by using the measured *α* in each case. The master curves have been shifted vertically for better comparison. In all cases, the data collapse is excellent. The lines are theoretical predictions, see main text. Inset: Collapse of reduced temperature profiles for the same conditions, and theoretical curves. In all cases, each curve for fixed *μ* contains 1280 points measured in 80 different simulations for varying *N, T*_0_ and *η*. The abscisas for the *μ* = 1.3 data have been divided by 4 to better visualize the results.

Figure 1 shows the temperature and density profiles measured for *μ* = 3 and varying *T*_0_, *η* and *N* (similar data are obtained for all other *μ*’s). These profiles are clearly nonlinear, and exhibit strong finite-size effects. However, the measured local density and temperature in each case are tightly coupled by the equilibrium EoS, *P* = *ρ*(*x)T*(*x*), with *P* the finite-size pressure measured in each simulation, see Fig. S2 and Section IIA in the Supplementary information, validating hypothesis (ii) above and confirming the robustness of MLE far from equilibrium^58^. Note that the thermal walls act as defects (akin to fixed, infinite-mass particles) which disrupt the structure of the surrounding fluid, defining two boundary layers where finite-size corrections mount up. To analyze below the fluid’s scaling behavior, we neglect data from these boundary layers (up to 7 cells adjacent to each wall), focusing the analysis on the remaining *bulk* profiles *ρ*(*x*) and *T*(*x*). For a given exponent *α*, each bulk density profile *ρ*(*x*) is then plotted as a function of *L*^−*α*^*ψx* (with 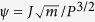 measured in each case, see Supplementary Fig. S3), and shifted by a constant *ζ* to achieve an optimal collapse among all scaled profiles, see Fig. 2a. The vector of optimal shifts ***ζ***_0_ for fixed *α* and *μ* is obtained by minimizing a standard collapse metric *D*(***ζ**; α, μ*) for the density profiles (defined in detail in Section III of the Supplementary information), which roughly speaking measures the relative average distance among all pairs of overlapping curves^59^, and the same shifts are used to collapse reduced temperature profiles, *T*(*x*)/*P*. The resulting data collapses are very sensitive to *α*, see Fig. 2b, so the the true anomaly exponent *α* can be measured with high precision for each mass ratio *μ* by minimizing *D*(*α, μ*) ≡ *D*(***ζ***_0_; *α, μ*) as a function of *α*. In fact, the distance function *D*(*α, μ*) has a pronounced minimum in *α* for each *μ*, see inset in Fig. 3a, whose width and depth allow to estimate the exponent error, see Supplemementary information, Section III.

Remarkably, the measured anomaly exponent is *non-universal*, depending non-monotonously on the mass ratio, *α* = *α*(*μ*), see Fig. 3a and Supplementary Table S1, growing first from small values at low *μ* to a maximum *α* ≈ 0.3 < 1/3 for *μ* = 2.2, and decaying afterwards to an asymptotic value *α* ≈ 1/4 for large *μ*. Figure 3b shows the master curves obtained from density and reduced temperature bulk profiles for different *μ*’s by using the measured *α*’s, and in all cases the resulting collapses are impressive, confirming that anomalous Fourier’s law (1)–(2) rules heat transport in this 1*d* model. Moreover, this surprising but unambiguous result also calls into question the prevailing conjecture that the anomaly in 1*d* heat transport is universal^6^,^23^,^24^,^25^,^26^,^27^,^28^,^29^,^30^.

**Figure 4:**
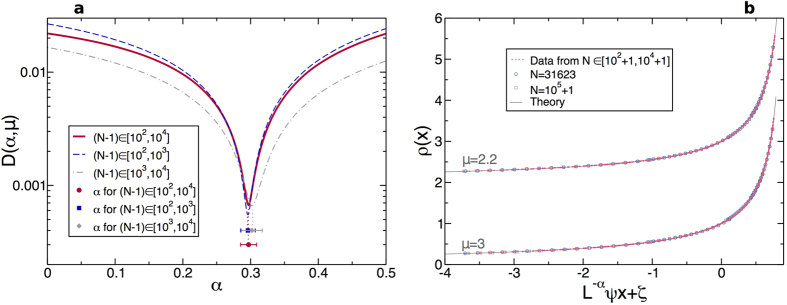
Ruling out finite-size corrections. (**a**) Distance metric *D*(*α, μ*) for *μ* = 3 as a function of *α* when considering all data, *N* ∈ [10^2^ + 1, 10^4^ + 1], *T*_0_ ∈ [2, 20], *η* ∈ [0.5, 3] (full line), and when *N* is restricted to small *N* ∈ [10^2^ + 1, 10^3^ + 1] (dashed line) or large *N* ∈ [10^3^ + 1, 10^4^ + 1] (dot-dashed line). Notice the logarithmic scale in the *y*-axis. The points and the errorbars below represent the estimated value of the anomaly exponent *α* in each case. Clearly, values of *α* obtained from the restricted sets in *N* are fully compatible between them and with the previous result using the combined sets, all points lying well within the errorbars. Note that the distance curve for large *N* is slightly wider than the small-*N* curve due to the somewhat larger uncertainties accompanying data for large *N*, a direct result of the strong growth of relaxation and correlation times with *N*, see Supplementary Fig. S4 and related discussion. (**b**) Collapse of density profiles for *μ* = 2.2 (top) and *μ* = 3 (bottom) obtained by using the measured anomaly exponent *α* in each case, see Supplementary Table S1. Small points correspond to the scaling collapse obtained for *N* ∈ [10^2^ + 1, 10^4^ + 1], *T*_0_ ∈ [2, 20], and *η* ∈ [0.5, 3], while bigger points correspond to additional results obtained from extensive simulations for larger system sizes, namely *N* = 31623 (○) and *N* = 10^5^ + 1 (□), with *T*_0_ = 20 and *η* ∈ [0.5, 3]. The line stands for the theoretical prediction, and the master curve for *μ* = 2.2 has been shifted vertically for better comparison.

At this point it is worth emphasizing that standard linear response methods to measure the heat conductivity typically yield an *effective* anomaly exponent in 1*d* which changes appreciably with the system size, 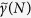, slowly converging to the asymptotic value *γ* at very large *N*^56^, see Section IIC in the Supplementary information. A natural question is hence whether the new anomaly exponent *α*(*μ*) measured with the novel scaling method introduced here exhibits similar finite-size corrections. A first clue that this is not the case is that, for *N* ∈ [10^2^ + 1, 10^4^ + 1], a slight change in the anomaly exponent measured with our scaling method completely destroys the observed collapse, see Fig. 2b, while the effective anomaly exponent measured with standard methods varies widely with *N* in the same *N*-range, e.g. 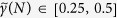, see Fig. 3b in ref. 56. In any case, in order to test quantitatively this idea, we divided our original data into two different subsets, one for small *N* ∈ [10^2^ + 1, 10^3^ + 1] and another one for large *N* ∈ [10^3^ + 1, 10^4^ + 1]. In this way both data subsets have the same amount of points, thus avoiding possible sampling issues. Next, we perform our scaling analysis on both subsets and obtain the collapse distance metric *D*(*α, μ*) as a function of *α* in each case. In both cases, small *N* vs large *N*, this function exhibits a pronounced minimum in *α* for each *μ*, and these minima identify the anomaly exponent as measured in each subset. Figure 4a shows the results of this analysis for mass ratio *μ* = 3, and the conclusion is clearcut: the anomaly exponents measured from the small-*N* and large-*N* subsets are fully compatible between them and with our previous measurement based on all *N* ∈ [10^2^ + 1, 10^4^ + 1], so no significant, systematic variation of the anomaly exponent with the system size is found beyond the stringent errorbars of our measurements. We found similar results for all other *μ*’s.

To further test the robustness of the measured anomaly exponents against order-of-magnitude changes in the system size, we also studied the steady-state heat transport in the diatomic hard-point fluid for *N* = 31623 and *N* = 10^5^ + 1, i.e. one order of magnitud beyond our previous simulations. The scale of these simulations is so large that we had to restrict the region of parameter space explored. In particular, we perform simulations of the aforementioned values of *N* for a large temperature gradient given by *T*_0_ = 20, global densities *η* = 0.5, 1, 2, 3, and two intermediate mass ratios *μ* = 3 and *μ* = 2.2 for which relaxation (and correlation) timescales are somewhat shorter (note that for both small and large mass ratios the fluid’s relaxation and correlation times increase drastically^60^,^61^). Figure 4b shows the collapse of density profiles for *μ* = 2.2 and *μ* = 3 obtained by using the measured anomaly exponent *α*(*μ*) in each case, namely *α*(*μ* = 2.2) = 0.308 and *α*(*μ* = 3) = 0.297, see Supplementary Table S1, once the new data for *N* = 31623 and *N* = 10^5^ + 1 have been added. In all cases the excellent collapse of all data for *N* ∈ [10^2^ + 1, 10^5^ + 1], i.e. across three orders of magnitude in the system size, strongly confirms the validity of the measured (non-universal) exponents in the large-*N* limit. Similar excellent collapses are also obtained for temperature profiles. Moreover, if a different anomaly exponent is used in the previous scaling plots (e.g. *α* = 1/3) no good collapse is obtained, as observed in e.g. Fig. 2b above, even if we restrict the plot to the largest values of *N*. These observations thus discard the possibility of a running anomaly exponent (at least within our stringent precision limits), demonstrating the robustness of the anomaly exponent *α* against order-of-magnitude changes in the system size and hence strengthening our conclusions.

**Figure 5:**
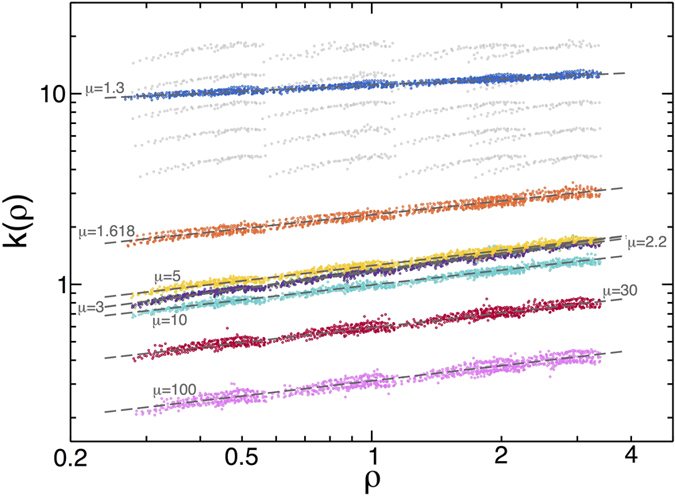
Density dependence of heat conductivity as captured by *k*(*ρ*). Light gray points show the curves obtained for *μ* = 3 before scaling data by *L*^−*α*^ along the *y*-axis, while dark color curves show the scaled curves for each *μ*. A power-law behavior is apparent in all cases. Dashed lines are power-law fits to the data, see main text and Supplementary Table S1.

We next focus on the density dependence of the heat conductivity 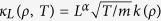. Interestingly, the dynamics of 1*d* hard-point fluids remains invariant under different scalings (of temperature, velocities, space, mass, etc.) ref. 5. Using such invariance, it is easy to show rigorously that 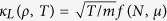, with *f* some adimensional function of *N* and *μ*. This in turn implies, via dimensional analysis, that necessarily *k*(*ρ*) = *aρ*^*α*^, with *a* some constant. This is fully confirmed in local measurements of the density dependence of the heat conductivity, from which we determine *a* = *a*(*μ*). Indeed, one can easily show from Eq. (2) that 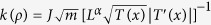, so for each set (*N, T*_0_, *η*) and fixed *μ* we performed discrete derivatives of the measured bulk temperature profile to evaluate *T*′(*x*) and plotted the previous expression, with *J* measured in each case, as a function of the associated *ρ*(*x*). Figure 5 shows the curves *k*(*ρ*) so obtained for different *μ*, which display the best collapse when the measured exponent *α*(*μ*) is used. Interestingly the resulting scaling functions, though somewhat noisy due to discretization effects, exhibit a clear power-law behavior, *k*(*ρ*) = *aρ*^*β*^, and the fitted exponent is fully compatible in all cases with the measured anomaly exponent, *β* = *α*(*μ*), see Fig. 3a above and Supplementary Table S1. These measurements thus prove in an independent way that the density dependence of the heat conductivity of the 1*d* diatomic hard-point gas does reflect the transport anomaly.

The above observation that *k*(*ρ*) = *aρ*^*α*^ opens the door to a full solution of the macroscopic heat transport problem for this model, see Section I of the Supplementary information. In particular, the universal master curve *F*(*u*) of Eq. (3) is


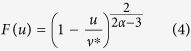


with 
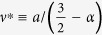
. This master curve depends on *μ* through the mass ratio dependence of *α* and *a*. Figure 3b displays the predicted master curves, with the only input of the measured *α*(*μ*) and *a*(*μ*), and the agreement with collapsed data is stunning in all cases. Closed forms for temperature profiles follow as


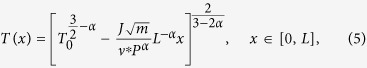


with density profiles given as *ρ*(*x*) = *P*/*T*(*x*), and *P* and *J* simply written in terms of external parameters *T*_0_, *T*_*L*_, *η*, and *L*, see Supplementary information, Section I. Note that this novel macroscopic solution is fully compatible with the known scaling symmetries of 1*d* hard-point fluids^5^. Interestingly, the master curve *F*(*u*) exhibits a vertical asymptote at *u* = ν*, see Eq. (4), implying the existence of a bound on the scaled current in terms of pressure,


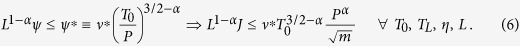


**Figure 6:**
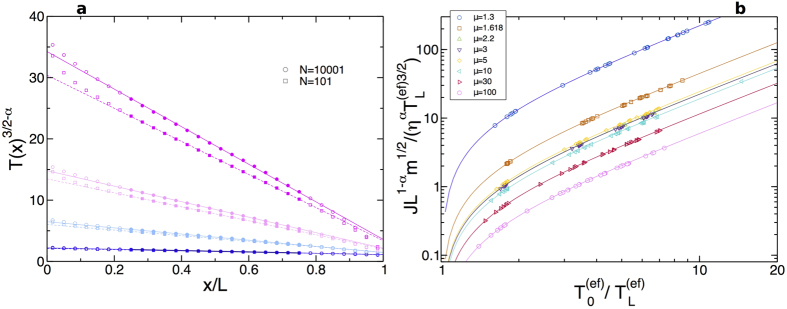
Testing additional predictions. (**a**) Measured temperature profiles to the power (3/2 − *α*) vs *x*, for *μ* = 3, *η* = 1, varying *T*_0_ ∈ [2, 20] and two different system sizes, *N* = 101 (□) and *N* = 10001 (○). Filled symbols correspond to the bulk, while open symbols signal the boundary layers. Lines have slope 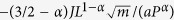, with *J* and *P* the measured current and pressure in each case, and the only fitting parameter corresponds to the *y*-intercept, which yields 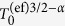 in each case. Note that 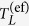 follows from 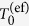 and the (fixed) slope. The agreement between lines and data confirm that *bulk* temperature (and density) profiles for any finite *N* are in fact those of a *macroscopic* diatomic hard-point gas sustaining a current *J* and a pressure *P* and subject to some effective *N*-dependent boundary conditions controlled by the boundary layers. For *μ* = 3, recall that *α* = 0.297(6) and *a* = 1.1633(9), see Supplementary Table S1. (**b**) Test of the macroscopic theory prediction for the heat current, see Eq. (8). For each mass ratio, 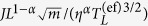 is plotted vs 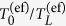, with *J* the measured current, and 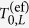 the effective boundary temperatures for bulk profiles measured in each case. The agreement between data (symbols) and theory (lines) is excellent in all cases.

Eq. (5) for temperature profiles can be readily tested against data. For that we plot *T*(*x*)^3/2−*α*^ vs *x*, with *T*(*x*) the measured temperature profiles for each *μ, N, η* and *T*_0_. This is predicted to be a straight line with slope 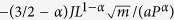, with *J* and *P* the measured current and pressure, respectively. Such linear dependence is confirmed for bulk temperature profiles in all cases (similar results hold also for density profiles), with the correct slope but with effective boundary temperatures (obtained from the *y*-intercept of the line) slightly different from the thermal wall temperatures in each case, 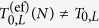. Figure 6a shows an example of this test for *μ* = 3, *η* = 1, varying *T*_0_ ∈ [2, 20] and two different system sizes, *N* = 101 (small) and *N* = 10001 (large), with excellent agreement in all cases. This shows that the measured *bulk* temperature (and density) profiles for any finite *N* are in fact those of a *macroscopic* diatomic hard-point gas sustaining a current *J* and a pressure *P* and obeying Eqs (1)–(2), 2, but subject to some effective *N*-dependent boundary conditions controlled by the boundary layers. Indeed, the striking collapse of data and the agreement with the macroscopic master curve in Fig. 3b strongly support this conclusion. This is a manifestation of the bulk-boundary decoupling phenomenon already reported in hard disks out of equilibrium^35^, which enforces the macroscopic laws on the bulk of the finite-sized fluid.

The effective boundary temperatures converge toward *T*_0,*L*_ as *N* increases, but at an exceedingly slow rate, 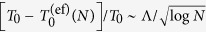 (see Supplementary Fig. S5), with Λ some amplitude, and this explains the persistent finite-size corrections found in the effective anomaly exponents measured with traditional linear response methods. Indeed, these methods approximate the heat conductivity as 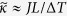, with Δ*T* = *T*_0_ − *T*_*L*_, and find that the so-defined empirical conductivity diverges as 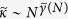 in 1*d*, with 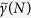 an *effective* anomaly exponent which exhibits itself persistent finite-size corrections^4^,^5^,^6^. Noting that the real temperature gradient driving the bulk fluid to sustain a current *J* is 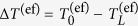 and taking into account the strong finite-size corrections affecting the boundary effective temperatures, it is easy to show (see Section IIC of the Supplementary information) that


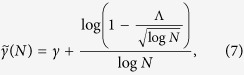


so the effective anomaly exponent 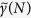 measured from the empirical conductivity 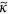 converges at an exceedingly slow rate toward the correct, asymptotic anomaly exponent *γ*, in a way that closely resembles actual measurements, see e.g. ref. 56. This confirms that the slowly-decaying (and artificial) *boundary* finite-size corrections associated to the boundary layers are responsible of the strong, persistent finite-size deviations affecting the effective anomaly exponent measured with the standard linear response method. Moreover, as our scaling method is independent of the boundary temperatures driving the system out of equilibrium, this explains why our results for the anomaly exponent *α* (that we conjecture is equal to *γ*) are free of these persistent finite-size corrections.

Finally, our macroscopic theory also offers a precise prediction for the heat current, see the Supplementary information, Section I. In particular, it predicts that 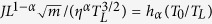, with *h*_*α*_(*z*) a well-defined function


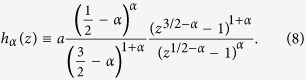


This prediction can be tested against data using the effective boundary temperatures 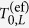 measured above, see Fig. 6b, and the agreement is excellent ∀*N, T*_0_, *η* for each *μ*.

## Discussion

Some comments are now in order. The excellent collapse of our data confirms that anomalous Fourier’s law (1) holds in this model with a well-defined (albeit size-dependent) conductivity functional 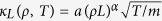. This is true even for finite *N* (as small as 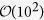
!) and under large temperature gradients, extending the range of validity of anomalous Fourier’s law deep into the nonlinear regime and evidencing the absence of higher-order (Burnett-like) corrections in 1*d*^35^.

In addition, we provide strong evidences supporting the breakdown of universality in anomalous Fourier’s law for 1*d* momentum-conserving systems^62^. In particular, we show with high accuracy that the new anomaly exponent *α* for the heat conductivity of the 1*d* diatomic hard-point fluid depends on the mass ratio *μ* between neighboring particles. This clear-cut observation, together with the conjectured equality between the different anomaly exponents, *α* = *γ*(=*δ*), calls into question the universality picture for heat transport based on renormalization-group and mode-coupling calculations^23^,^24^. Note however that our results do not say anything about or contradict the Lévy/KPZ universality of the equilibrium time correlators of the *conserved* (hydrodynamic) fields, recently predicted within nonlinear fluctuating hydrodynamics and tested in simulations^25^,^26^,^27^,^28^.

Different tests of the universality conjecture for the heat transport anomaly have been performed in the past for the diatomic hard-point fluid using a number of methods, including both nonequilibrium simulations of heat transport in the linear response regime and equilibrium measurements of current time-correlation functions^4^,^5^,^6^. All tests confirm the existence of the heat transport anomaly for this model. However, the accuracy of the standard methods to determine the anomaly exponents is severely hampered by the strong finite-size corrections affecting these measurements, making very difficult to discern the breakdown of universality here reported. For instance, determining the heat conductivity via the standard nonequilibrium route leads to a running effective anomaly exponent 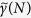 which exhibits itself persistent finite-size deviations and poor convergence with *N*^56^. Our scaling results explain the origin of this extremely slow convergence, which in brief can be traced back to the mixing of the artificial but very strong *boundary* finite-size corrections with the most important *bulk* scaling behavior. Since our collapse procedure is independent of the boundary driving, this explains why our scaling results for the anomaly exponent *α* are free of these persistent finite-size effects, offering very precise measurements which remain robust across three decades in *N*. On the other hand, the standard equilibrium (Green-Kubo) route to study the anomaly can typically test the *compatibility* of the long-time tail exponent *δ* with the universality prediction, but cannot discriminate in most cases the small exponent differences associated to the universality violation here reported. This is particularly relevant for mass ratio *μ* = 3, for which most equilibrium tests have been performed and where our scaling results yield an anomaly exponent close to (but different from) 1/3, the universality prediction for this model. Therefore it would be desirable to perform standard equilibrium tests also for other mass ratios for which the difference between the universality exponent and the one we measure from scaling are more definite, as e.g. *μ* = 10 for which *α* = 0.260(14), see Supplementary Table S1. We note however that some recent and very precise simulations of the equilibrium diatomic hard-point fluid for *μ* = 3 and *N* = 4096 suggest^28^ an equilibrium anomaly exponent *δ* = 0.33 > *α*(*μ* = 3) = 0.297(6). This apparent discrepancy, which needs further investigation, could mean that the relation between the different anomaly exponents is not as straightforward as conjectured.

Which is the origin of the universality breakdown here reported? This violation of universality may hint at the possible existence of hidden slowly-evolving fields in the diatomic hard-point gas other than the standard (locally-conserved) hydrodynamic ones. Remarkably, such intriguing behavior has been already reported in the nonequilibrium response of this model to a shock wave excitation^36^,^37^, and suggests that a more convoluted fluctuating hydrodynamics description (including the additional slow fields, as in granular fluids^63^) may be needed to understand anomalous transport in this model. Moreover, as recently put forward^29^, the existence of further slowly-evolving fields may give rise to an infinite discrete (Fibonacci) family of anomaly exponents that can coexist in different regions of parameter space for a given model^29^, changing from one value to another as a control parameter is varied, a behavior reminiscent of our results.

The question remains as to how to reconcile the local nature of Fourier’s law with the non-local *L*^*α*^-term in *κ*_*L*_(*ρ, T*). Our data suggest that this could be achieved in a nonlinear fluctuating hydrodynamics description of the problem derived via an anomalous, non-diffusive hydrodynamic scaling of microscopic spatiotemporal variables, *x* → *x*/*L*^1−*α*^ and *t* → *t*/*L*^2−3*α*^. We also mention that recent results suggest yet another mesoscopic description of anomalous transport in 1*d* in terms of fractional diffusion equations and/or heat carriers with Lévy-walk statistics^43^,^64^,^65^,^66^. As far as we know, this description does not seem compatible with the scaling and data collapses observed in this work. Finally, it would be interesting to apply the scaling method here developed to other paradigmatic models of heat transport in low dimensions, as e.g. the Fermi-Pasta-Ulam model of anharmonic oscillators and the hard-square or -shoulder potentials^3^,^4^,^5^,^6^, where the reported universality breakdown can be further investigated. The role of conservative noise^65^,^66^ as a smoothing mechanism to get rid of non-hydrodynamic, hidden slow fields should be also investigated.

## Additional Information

**How to cite this article**: Hurtado, P. I. and Garrido, P. L. A violation of universality in anomalous Fourier’s law. *Sci. Rep.* **6**, 38823; doi: 10.1038/srep38823 (2016).

**Publisher's note:** Springer Nature remains neutral with regard to jurisdictional claims in published maps and institutional affiliations.

## Supplementary Material

Supplementary Information
