# Supplementary material for: A violation of universality in anomalous Fourier’s law
Source: Sci Rep. 2016 Dec 13;6:38823. doi: 10.1038/srep38823 (PMC5153844; doi:10.1038/srep38823)
Supplement: Supplementary Information [file srep38823-s1.pdf]

Supplementary Information: A violation of universality in anomalous Fourier's law

Pablo I. Hurtado* and Pedro L. Garrido†
Institute Carlos I for Theoretical and Computational Physics and Departamento de Electromagnetismo y Física de la Materia, Universidad de Granada, 18071 Granada, Spain
(Dated: November 11, 2016)

I. SCALING IN FOURIER'S LAW

In this Section we will show that the stationary density and temperature profiles of the $1d$ diatomic hard-point fluid driven out of equilibrium by an arbitrary temperature gradient follow from an universal master curve, provided that three simple hypotheses hold (see below). It will be then trivial to show that the reverse statement also holds true, i.e. that a nonequilibrium $1d$ fluid whose density and temperature profiles collapse onto an universal master curve is bounded to obey the three mentioned properties. These hypotheses are:

(i) **Fourier's law:** In the steady state, the nonequilibrium fluid sustains a non-vanishing heat current J proportional to the temperature gradient

$$J = -\kappa_L(\rho, T)\frac{dT(x)}{dx}\,, \quad x \in [0, L]\,, \tag{S1}$$

with $\kappa_L(\rho, T)$ a well-defined local conductivity functional which may depend on L.

(ii) **Macroscopic local equilibrium (MLE):** This amounts to assume that local thermodynamic equilibrium holds at the macroscopic level, in the sense that the local density and temperature are related by the *equilibrium* equation of state (EoS) [1]. For the $1d$ diatomic hard-point gas studied in this paper, it is simply the ideal gas EoS

$$P = \rho T\,, \tag{S2}$$

with P the fluid's pressure.

(iii) **Heat conductivity scaling:** Due to the homogeneity of the interaction potential, the heat conductivity of the $1d$ diatomic hard-point gas exhibits a well-known density temperature separability [2]. Moreover, standard dimensional analysis arguments show that $\kappa \propto \sqrt{T/m}$ [2], and the known dimensional anomaly for transport implies in turn that $\kappa \propto L^\alpha$ at leading order. We now raise these arguments to a formal scaling ansatz

$$\kappa_L(\rho, T) = L^\alpha \sqrt{T/m} k(\rho)\,, \tag{S3}$$

with $k(\rho)$ a function solely dependent on density. Note that this ansatz discards possible subleading corrections in L.

We may now use the MLE property (ii) and the conductivity scaling ansatz (iii) to write Fourier's law in terms only of the density field. In particular, using the EoS to write $T(x) = P/\rho(x)$, we obtain

$$\frac{J\sqrt{m}}{P^{3/2}} L^{-\alpha} = G'(\rho)\frac{d\rho}{dx} = \frac{dG(\rho)}{dx}\,, \tag{S4}$$

where $G'(\rho) \equiv k(\rho)\rho^{-5/2}$ and $'$ denotes derivative with respect to the argument. This equation, together with the boundary conditions for the density field, $\rho(x = 0, L) = \rho_{0,L}$, which can be inferred from the constraints

$$\frac{T_0}{T_L} = \frac{\rho_L}{\rho_0}\,, \tag{S5}$$

$$\eta = \frac{1}{L}\int_0^L \rho(x)dx = \frac{\displaystyle\int_{\rho_0}^{\rho_L} \rho\, G'(\rho)d\rho}{G(\rho_L) - G(\rho_0)}\,, \tag{S6}$$

completely define the macroscopic problem in terms of $\rho(x)$. Note that the externally controlled parameters in the problem are the temperatures of the boundary reservoirs, $T_{0,L}$, and the global number density η. The pressure and the heat current can be now obtained as $P = T_0\,\rho_0$ and $J = P^{3/2}[G(\rho_L) - G(\rho_0)]/(L^{1-\alpha}\sqrt{m})$.

A simple yet striking consequence of hypotheses (i)-(iii) can be now directly inferred from Eq. (S4). In fact, as both J and P are state-dependent constants, this immediately implies that $G[\rho(x)] = \psi L^{-\alpha} x + \zeta$, i.e. $G[\rho(x)]$ is a linear function of position $x \in [0, L]$, with $\psi = J\sqrt{m}/P^{3/2}$ the reduced current and $\zeta = G(\rho_0)$ a constant. Equivalently,

$$\rho(x) = F\left(\frac{\psi}{L^\alpha}x + \zeta\right)\,, \tag{S7}$$

where we have assumed that the function $G(\rho)$ has a well-defined inverse $F(u) \equiv G^{-1}(u)$. This assumption seems reasonable as steady density profiles are typically well behaved and readily measurable in simulations and experiments, see e.g. Fig. 1 in the main text. Therefore, according to Eq. (S7), there exists a single universal master curve $F(u)$ from which any steady state density profile follows after a linear spatial scaling $x = L^\alpha(u - \zeta)/\psi$. This scaling behavior is automatically transferred to temperature profiles via the local EoS $P = \rho(x)T(x)$, so

$$\frac{T(x)}{P} = \frac{1}{F\left(\frac{\psi}{L^\alpha}x + \zeta\right)}\,. \tag{S8}$$

* phurtado@onsager.ugr.es
† garrido@onsager.ugr.es

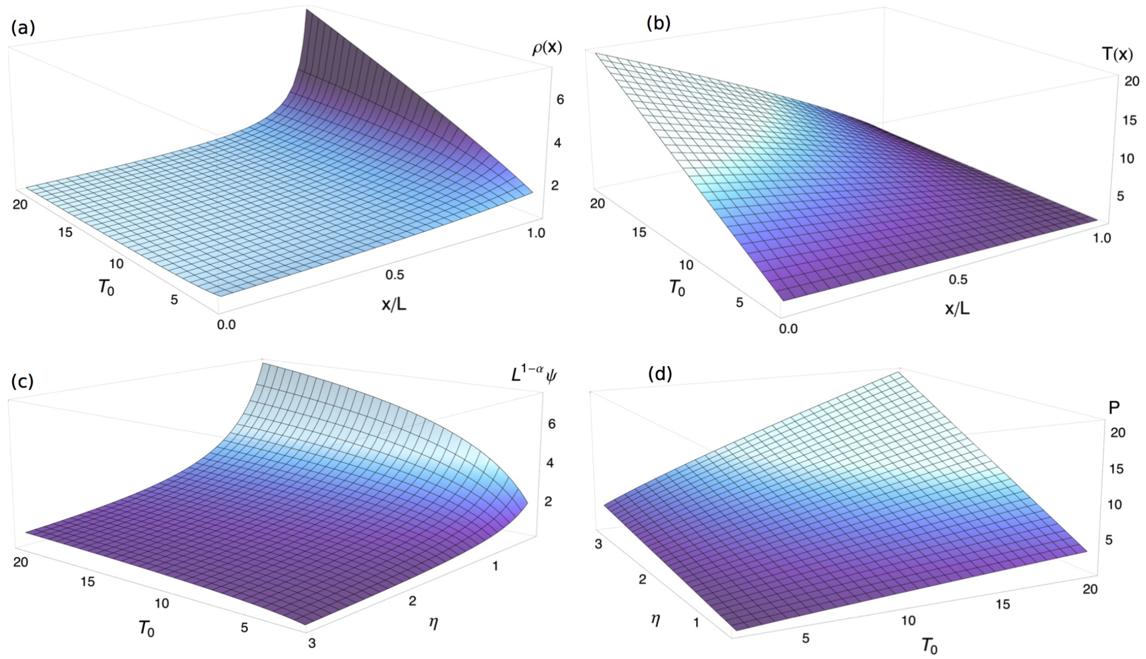

FIG. S1. **Theoretical predictions.** Density (a) and temperature (b) profiles as a function of T_0 for $\eta = 1$, obtained from the full solution of the macroscopic heat transport problem for the $1d$ diatomic hard-point gas, see Eq. (S10) and the associated discussion. Also shown are the η- and T_0-dependence of (c) the scaled reduced current, $L^{1-\alpha}\psi$, and (d) the nonequilibrium fluid's pressure P, see Eqs. (S12)-(S13). All these curves are for a mass ratio $\mu = 3$, for which $\alpha = 0.297(6)$ and $a = 1.1633(9)$, see Table S1 below where all measured anomaly exponents for different μ's can be found.

These scaling laws are independent of the global density η or the nonequilibrium driving defined by the baths temperatures T_0 and T_L, depending exclusively on the function $k(\rho)$ controlling the fluid's heat conductivity. Alternatively, Eq. (S7) implies that any measured steady density profile can be collapsed onto the universal master curve $F(u)$ by scaling space by the scaled reduced current $L^{-\alpha}J\sqrt{m}/P^{3/2}$ measured in each case and shifting the resulting profile an arbitrary constant ζ (similarly for temperature profiles). This suggests a simple scaling method to obtain the universal master curves in simulations or experiments, a procedure that we implement in the main text. Note that these results are not limited to the $1d$ diatomic hard-point gas; equivalent results hold for general d-dimensional (non-critical) fluids driven arbitrarily far from equilibrium, see Ref. [2] for a proof.

Proving the reverse statement, i.e. that a $1d$ fluid obeying Eqs. (S7)-(S8) does fulfill also properties (i)-(iii) above, is now trivial. In particular, the MLE property (ii) is automatically satisfied by construction. Moreover, inverting the scaling in (S7) to obtain $G[\rho(x)]$ and differentiating this functional with respect to x we arrive at $J = -L^{\alpha}\sqrt{T/m}G'(\rho)\rho^{5/2}T'(x)$, where we used that $T(x) = P/\rho(x)$, see Eqs. (S7)-(S8). This hence proves that properties (i) and (iii) also hold, with a heat conductivity given by Eq. (S3) with $k(\rho) = G'(\rho)\rho^{5/2}$.

The combination of our scaling ansatz for the heat conductivity and well-known dynamical invariances of $1d$ hard point fluids under scaling of different magnitudes (as e.g. temperature, velocities, mass, space, etc.) results in a well-defined density dependence for the heat conductivity, see main text, namely $k(\rho) = a\rho^{\alpha}$, with a a constant of $\mathcal{O}(1)$. Such power-law dependence, which reflects the transport anomaly, is fully confirmed in local measurements of the density dependence of κ_L, see Fig. 5 in the main text, from which we obtain precise estimates of the amplitude $a(\mu)$, see Table S1. Such clear-cut observation, together with the scaling formalism described above, allows now for a complete solution of the *macroscopic* transport problem for this model, written in terms of the external control parameters, namely T_0, T_L, η and L, together with α and a. In fact, recalling that $G'(\rho) = k(\rho)\rho^{-5/2}$ we obtain that $G(\rho) = \nu^*(1-\rho^{\alpha-3/2})$, with $\nu^* \equiv a/(\frac{3}{2}-\alpha)$ and where we have chosen an arbitrary constant such that $F(0) = 1 = G^{-1}(0)$. The universal master curve hence reads

$$F(u) = (1-\frac{u}{\nu^*})^{\frac{2}{2\alpha-3}} . \qquad \text{(S9)}$$

This prediction is compared with the measured master curves in Fig. 3 (right panel) of the main text, and the agreement is excellent for all mass ratios μ. Eq. (S9) implies in turn that density profiles can be written as

$$\rho(x) = \left[\left(\frac{P}{T_0}\right)^{\alpha-\frac{3}{2}} - \frac{\psi}{\nu^*}L^{-\alpha}x\right]^{\frac{2}{2\alpha-3}} , \qquad \text{(S10)}$$

while temperature profiles simply follow from $T(x) = P/\rho(x)$, namely

$$T(x) = \left[T_0^{\frac{3}{2}-\alpha} - \frac{J\sqrt{m}}{\nu^* P^{\alpha}}L^{-\alpha}x\right]^{\frac{2}{3-2\alpha}} . \qquad \text{(S11)}$$

μ	α	β	a
1.3	0.108 (9)	0.109 (1)	11.105 (8)
1.618	0.242 (23)	0.2408 (18)	2.307 (3)
2.2	0.308 (5)	0.3068 (11)	1.1765 (9)
3	0.297 (6)	0.2964 (11)	1.1633 (9)
5	0.266 (11)	0.2641 (12)	1.2622 (12)
10	0.260 (14)	0.2632 (19)	0.9874 (14)
30	0.258 (18)	0.257 (1)	0.5942 (12)
100	0.265 (22)	0.2648 (23)	0.3095 (5)

TABLE S1. **Anomaly exponents.** Measured anomaly exponents α and their error for different mass ratios μ, see Fig. 3 in main paper. Also shown are the fitted exponent and amplitude of the power-law density dependence of the conductivity, $k(\rho) = a\rho^\beta$, see Fig. 5 in the main text. Notice that in all cases $\beta = \alpha$ within error bars, as predicted.

The calculation is completed by expressing the heat current J and the pressure P in terms of the external parameters by using Eqs. (S5)-(S6) above. This yields

$$P = \eta \left(\frac{\frac{1}{2}-\alpha}{\frac{3}{2}-\alpha} \right) \left(\frac{T_0^{3/2-\alpha} - T_L^{3/2-\alpha}}{T_0^{1/2-\alpha} - T_L^{1/2-\alpha}} \right) , \tag{S12}$$

$$J = \frac{a\eta^\alpha (\frac{1}{2}-\alpha)^\alpha}{L^{1-\alpha}\sqrt{m}(\frac{3}{2}-\alpha)^{1+\alpha}} \frac{(T_0^{3/2-\alpha} - T_L^{3/2-\alpha})^{1+\alpha}}{(T_0^{1/2-\alpha} - T_L^{1/2-\alpha})^\alpha} \tag{S13}$$

The last equation for the current can be rewritten as $J = \eta^\alpha L^{\alpha-1} m^{-1/2} T_L^{3/2} h_\alpha(T_0/T_L)$, with

$$h_\alpha(z) \equiv a \frac{(\frac{1}{2}-\alpha)^\alpha}{(\frac{3}{2}-\alpha)^{1+\alpha}} \frac{(z^{3/2-\alpha}-1)^{1+\alpha}}{(z^{1/2-\alpha}-1)^\alpha} . \tag{S14}$$

These predictions are fully confirmed by simulations data, see Fig. 6 in main text and Section II below. As a self-consistent check, note that in the equilibrium limit $T_0 \to T_L$ both the pressure and the heat current converge to their expected value, namely $P = \eta T_L$ and $J = 0$. Fig. S1 shows the density and temperature profiles predicted for a *macroscopic* diatomic hard-point fluid as a function of T_0 for $\eta = 1$, as well as the pressure and the scaled reduced current $L^{-\alpha}\psi$ as a function of T_0 and η. These plots are obtained for a particular mass ratio $\mu = 3$, for which $\alpha = 0.297(6)$ and $a = 1.1633(9)$, see Table S1, and yield an excellent comparison with simulation data, see Fig. 1 in the main text and Fig. S3 in Section II.

Interestingly, the master curve $F(u)$ obtained above exhibits a vertical asymptote at $u = \nu^*$, see Eq. (S9), and this implies in turn the existence of a maximal scaled reduced current ψ^*. Indeed, for the associated density profile to exist in its whole domain $x \in [0, L]$, see Eq. (S10), the following condition must hold

$$\psi \leq \frac{\nu^*}{L^{1-\alpha}} \left(\frac{T_0}{P} \right)^{3/2-\alpha} \equiv \frac{\psi^*}{L^{1-\alpha}} , \tag{S15}$$

with P expressed as in Eq. (S12). This defines a maximal scaled reduced current ψ^*, such that the scaled current $L^{1-\alpha}J \leq \psi^* P^{3/2}/\sqrt{m} = \nu^* T_0^{3/2-\alpha} P^\alpha/\sqrt{m}$ $\forall\, T_0, T_L, \eta, L$, defining an upper bound on the heat current in terms of the nonequilibrium pressure. The maximal scaled reduced current increases monotonously with T_0, saturating to an asymptotic value in the $T_0 \to \infty$ limit, namely

$$\psi^* \xrightarrow[T_0\to\infty]{} \frac{a\,(\frac{3}{2}-\alpha)^{1/2-\alpha}}{[\eta\,(\frac{1}{2}-\alpha)]^{3/2-\alpha}} . \tag{S16}$$

Note however that both $L^{1-\alpha}J$ and P diverge as $T_0 \to \infty$, though ψ^* remains finite.

To end this section, we remark that Eqs. (S9)-(S13) constitute the solution of the *macroscopic* transport problem for this model. A comparison of the predicted density and temperature profiles, see Eqs. (S10)-(S11), with the finite-size data of Fig. 1 in the main text allow us to investigate in the main text the bulk-boundary decoupling phenomenon in detail by quantifying the jump between the effective boundary conditions imposed by the boundary layers on the bulk fluid and the empirical bath temperatures.

II. ADDITIONAL RESULTS

In this Section we provide additional data, obtained from our extensive simulations of the 1d diatomic hard-point fluid model, which support our conclusions in the main text.

A. Macroscopic local equilibrium, pressure and reduced current

Our first goal is to test the macroscopic local equilibrium (MLE) property directly from our data. As described above, MLE conjectures that local thermodynamic equilibrium holds at the macroscopic level, in the sense that the stationary density and temperature fields are locally related by the *equilibrium* equation of state (EoS) [1], which for this model is simply the ideal gas EoS,

$$P = \rho(x)T(x) .$$

In order to test MLE, we hence take the density and temperature profiles of Fig. 1 of main text measured for $\mu = 3$ and different T_0, N, η, and plot in Fig. S2 the local reduced temperature, $T(x)/P$, with P the finite-size pressure measured in each simulation (see below), as a function of the associated local density $\rho(x)$. All data, comprising 2400 points from 80 different simulations for widely different systems sizes, temperature gradients and global densities, collapse onto a single curve which follows with high precision the expected ideal-gas behavior $1/\rho$, see line in Fig. S2 and inset therein. Note that, interestingly, the excellent data collapse is maintained also for points within the boundary layers near the thermal walls. Moreover, similar results hold for all mass

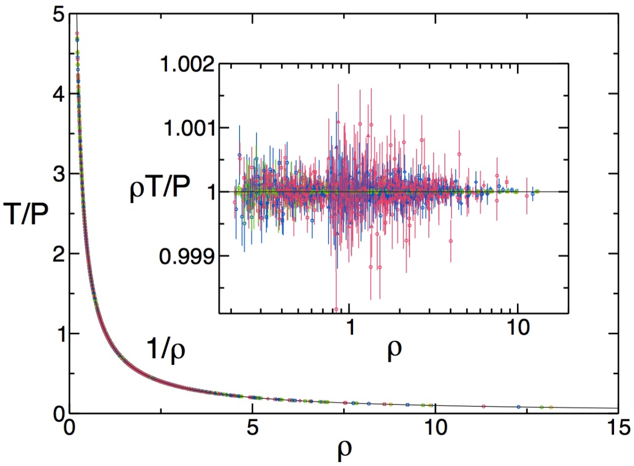

FIG. S2. **Macroscopic local equilibrium.** Measured local reduced temperature, $T(x)/P$, plotted as a function of the associated local density $\rho(x)$ for $\mu = 3$ and $\forall T_0, \eta, N$, corresponding to all profiles displayed in Fig. 1 of the paper and summing up to 2400 data points from 80 different simulations. An excellent data collapse is obtained which follows with high precision the expected ideal-gas behavior $1/\rho$, plotted as a thin line. Inset: Scaling plot of $\rho(x)T(x)/P$ vs $\rho(x)$ for the same conditions. These data show that macroscopic local equilibrium is a very robust property, even in the presence of strong finite-size corrections on the hydrodynamic profiles.

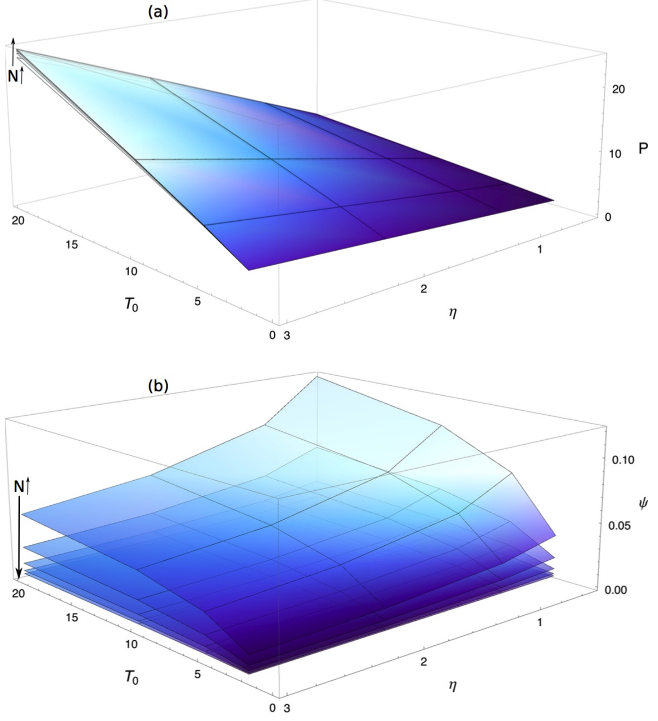

FIG. S3. **Pressure and reduced current.** Measured pressure P (a) and reduced current $\psi = J\sqrt{m}/P^{3/2}$ (b) as a function of T_0 and η for $\mu = 3$ and different system sizes N. Data here refer to wall observables, though the associated bulk observables yield completely equivalent results.

ratios μ studied in this paper. In this way, the observed high-precision data collapses confirm the robustness of the MLE property far from equilibrium [1], even in the presence of important finite size effects, validating in an independent manner one of the hypotheses underlying the scaling picture of Section I.

We next focus on the nonequilibrium fluid's pressure P and the heat current J flowing through the system, that we measure both in the bulk and at the thermal walls. These observables are necessary in order to scale the spatial coordinate of the hydrodynamic profiles using the measured reduced current $\psi = J\sqrt{m}/P^{3/2}$ in each case. Fig. S3 shows the measured P (a) and ψ (b) as a function of T_0 and η for $\mu = 3$ and different system sizes N. These data refer to wall observables, though the associated bulk observables yield completely equivalent results (as otherwise expected). The comparison of these data with our predictions in Section I is excellent, see Fig. S1 above.

B. Slow relaxation in $1d$ transport

In order to test the robustness of the measured anomaly exponents against order-of-magnitude changes in the system size, we have made a considerable computational effort to characterize the steady-state heat transport in the diatomic hard-point fluid for very large N's, namely $N = 31623$ and $N = 10^5 + 1$ (see main text). Of course it would be desirable to go even beyond $N = 10^5 + 1$. However, it is important to note that for such very large values of N obtaining *reliable* results from simulations of $1d$ heat transport is exceedingly difficult. The underlying reason is not only that more particles need more computer time to simulate, but also that relaxation (and correlation) times increase greatly with N. This is due to the appearance of current (and momentum) waves in $1d$ which bounce back and forth between the thermal walls at a constant and well-defined speed while they are slowly damped away, a result of the dimensional constraint which strongly suppress local fluctuations. As far as we know, this remarkably slow relaxation mechanism has not been described yet in the literature on $1d$ transport, so we add details about the relaxation process and timescales next.

In particular, we have performed a large number of relaxation experiments for different values of $N \in [10^2 + 1, 10^4 + 1]$, for $\mu = 3$, $T_0 = 20$ and $\eta = 1$. Initial states for these experiments are randomly drawn from a local equilibrium measure corresponding to the macroscopic density and temperature profiles (obtained from extensive simulations for intermediate system sizes after a long empirical relaxation time). These initial states hence display on average the stationary density and temperature stationary profiles, but lack the weak but long-range correlations which characterize any nonequilibrium steady state (and which are in fact responsible for heat trans-

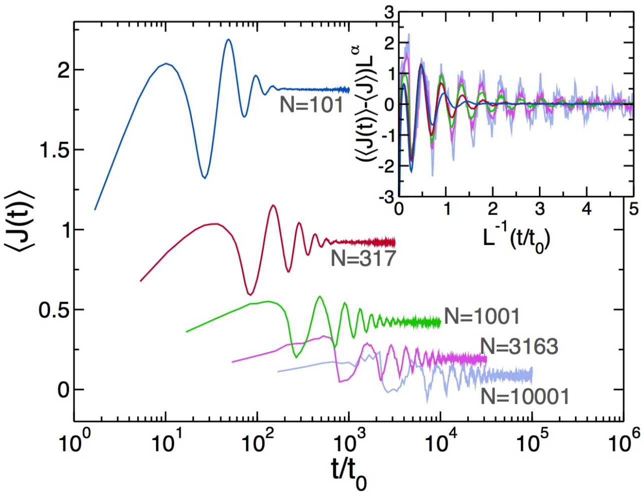

FIG. S4. **Slow relaxation in** $1d$ **transport.** Relaxation of the average instantaneous current as a function of time (measured in units of the mean free time of a heavy particle in a cold environment) for $\mu = 3$, $T_0 = 20$, $\eta = 1$, and different values of $N \in [10^2 + 1, 10^4 + 1]$. Notice the logarithmic scale in the time axis. Relaxation to the nonequilibrium steady state proceeds via a damped current wave with a period which increases linearly with the system length L. Inset: Scaling plot of the damped current wave.

port). To characterize the relaxation process to the true nonequilibrium steady state, we measured the average instantaneous energy current as a function of time,

$$\langle J(t)\rangle \equiv \frac{1}{2L}\langle \sum_{i=1}^{N} m_i v_i(t)^3\rangle \,,$$

with the average taken over many different realizations of the relaxation process starting from the random initial states defined previously. Fig. S4 shows the relaxation of $\langle J(t)\rangle$ for different N as a function of time, measured in units of $t_0 = \sqrt{M/(2T_L\eta^2)}$, the mean free time of a heavy particle in a cold environment. Note the logarithmic scale in the time axis. From this figure it is clear that the building of the faint but long-range correlations associated to the nonequilibrium steady state proceeds via the formation of a current wave which bounces back and forth between the thermal walls at a constant velocity, while being slowly damped in the nonequilibrium fluid. This damped current wave is accompanied by a similar momentum wave. The period of these waves scales linearly with the system size, and hence the relaxation time to reach the steady state also grows linearly with L. In fact, by scaling time by L^{-1} and the excess current by L^{α}, with $\alpha(\mu = 3) = 0.297$, a good collapse is obtained (at least for large N), see inset to Fig. S4, which suggest that

$$\langle J(t)\rangle = \langle J\rangle + L^{-\alpha}\mathcal{G}\left(\frac{t}{Lt_0}\right) \,,$$

with $\mathcal{G}(\tau)$ some scaling function.

The most relevant point here is that relaxation (and correlation) times grow linearly with the system size, making very difficult to obtain good statistics of transport for large enough N. In fact, the computer time needed to perform one collision per particle on average in an optimized event-driven molecular dynamics simulation of N particles scales as $(N \log N) \times \tau$ (due to the cost of re-ordering the collision time queue in a heap data structure), with $\tau \sim 10^{-5}\,s$ the typical timescale of an elementary event. As the fluid relaxation and correlation times scale linearly with N, the computer time needed to obtain reliable data averages hence scales as $t_{\rm sim} \sim n_{\rm exp} \times (N^2 \log N) \times \tau$, with $n_{\rm exp}$ the number of measurements to obtain good statistics. For $N = 10^5$ and $n_{\rm exp}$ in the few hundreds (at least), we thus have the mind-blowing timescale $t_{\rm sim} \sim 10^8\,s$, right at the edge of modern-day multiprocessor computer power. For these reasons going beyond $N \sim 10^5$ does not seem feasible at this moment.

C. Finite-size corrections for the effective anomaly exponent measured with standard methods

One of the most popular methods to measure the heat conductivity of a $1d$ fluid and characterize along the way the associated anomaly exponent consists in setting the model fluid with N particles and density η under a small temperature gradient, with fixed wall temperatures $T_{0,L}$, and then increasing N at constant density. For large enough N the overall temperature gradient is small enough so one can *approximate* Fourier's law as

$$J = -\kappa_L(\rho, T)\frac{dT}{dx} \approx +\tilde{\kappa}\frac{\Delta T}{L} \,, \qquad \text{(S17)}$$

with $\Delta T = T_0 - T_L$, J the measured current and $L = N/\eta$. In this way, the estimated heat conductivity follows as $\tilde{\kappa} \approx JL/\Delta T$, which is expected to diverge as N^{γ} for *large enough* values of N (though there is no way of knowing a priori which value of N is *large enough*). What it's typically found however in actual, cutting-edge simulations is an effective heat conductivity diverging as $\tilde{\kappa} \sim N^{\tilde{\gamma}(N)}$, with an *effective* anomaly exponent which exhibits itself persistent finite-size corrections [3].

Indeed, this approximate method completely neglects the nonlinear density and temperature dependence of the heat conductivity, and by construction it can only yield meaningful results in the limit $N \to \infty$. It is therefore no surprise that the effective anomaly exponent derived within this approach for finite N varies slowly with the system size, as in fact the very definition of $\tilde{\kappa}$ above (and hence γ) is correct only asymptotically. This weakness in the above definition is reinforced by the fact that, for a given N, the heat conductivity measured in this way differs from estimations of κ using alternative approaches, as e.g. the also popular Green-Kubo equilibrium method [3] (which, by the way, is again exact only in the $N \to \infty$ limit). Moreover, not only the estimated value of κ for a

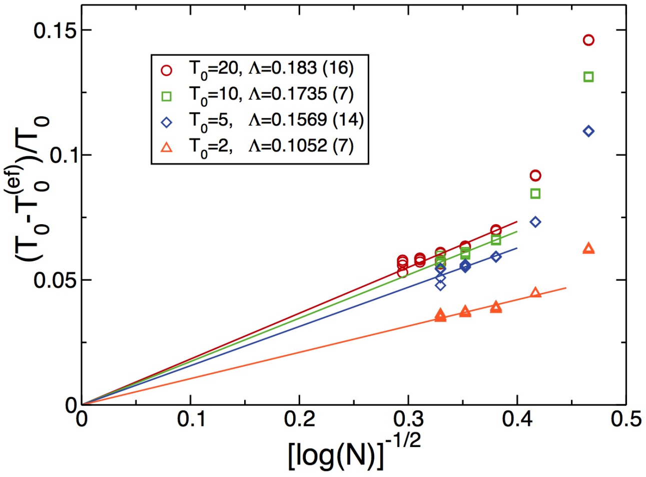

FIG. S5. **Decay of finite-size corrections for the boundary effective temperature.** The relative temperature gap at the hot thermal wall, defined as $(T_0 - T_0^{(\mathrm{ef})})/T_0$, as a function of $1/\sqrt{\log N}$ for $\mu = 3$ and different values of $T_0 \in [2, 20]$ and $\eta \in [0.5, 3]$. Our data are compatible with a linear decay for large enough N, $(T_0 - T_0^{(\mathrm{ef})})/T_0 \sim \Lambda/\sqrt{\log N}$, see lines, with Λ a small amplitude. This extremely slow, $1/\sqrt{\log N}$ decay of boundary finite-size corrections explains the running effective anomaly exponents previously reported in literature.

given N differs among different approaches, but also its scaling with N, and hence the estimation of the anomaly exponent.

The overall situation is therefore rather unsatisfactory, making extremely difficult to characterize reliably and accurately anomalous Fourier's law in $1d$ with these standard linear response methods. In fact, when measuring γ with standard methods one needs to reach huge system sizes, as large as $N \sim 10^5$, to appreciate certain convergence, and even in this case the asymptotic behavior is not yet clearly defined, see e.g. Ref. [3].

In contrast with the standard methods described above, our scaling approach takes full advantage of the nonlinear character of the heat conduction problem and provides a fully consistent and highly accurate description of all measured data in a broad range of parameters, including three orders of magnitude in $N \in [10^2 + 1, 10^5 + 1]$, but also a wide range of temperature gradients (from the linear response regime to the fully nonlinear domain) and densities. The new anomaly exponent $\alpha(\mu)$ that we conjecture equal to γ and is measured from the striking collapse of large amounts of data is well-defined, exceptionally robust and does not change with the system size in the broad range explored (see analysis in the main text). This contrasts with the running, effective exponent obtained from the standard linear response methods described above, which varies widely (and far beyond our precision limits) in the same N-range, i.e. $\tilde{\gamma}(N) \in [0.25, 0.5]$, see e.g. Fig. 3.b in Ref. [3].

A natural question now is: why do standard methods measure an effective anomaly exponent which converges so slowly with N? The answer to this question lies at the bulk-boundary decoupling phenomenon reported in this work (see main text), i.e. the fact that the bulk of the finite-size nonequilibrium fluid behaves as a macroscopic, infinite system subject to some *effective* boundary temperatures, $T_{0,L}^{(\mathrm{ef})}(N)$. Indeed, we measured and characterized the effective $T_{0,L}^{(\mathrm{ef})}$ $\forall \mu, N, \eta$ and T_0 by comparing the measured temperature profiles with the theoretical prediction based on our scaling theory, see left panel of Fig. 6 in the main text and the associated discussion. The effective boundary temperatures so obtained turn out to be N-dependent, slightly differing from the wall temperatures $T_{0,L}$ but converging to these values as N increases. Most surprisingly, however, this convergence is exceedingly slow. In fact, Fig. S5 shows the measured relative temperature gap at the hot thermal wall, defined as $(T_0 - T_0^{(\mathrm{ef})})/T_0$, as a function of $1/\sqrt{\log N}$ for $\mu = 3$ and different values of $T_0 \in [2, 20]$ and $\eta \in [0.5, 3]$, including data for $N = 31623$ and $N = 10^5 + 1$ obtained for $T_0 = 20$. For large enough N, namely $N \geq 10^3$ (or even $N \geq 317$ for small T_0), our data in Fig. S5 are compatible with a linear law, hence implying a decay of the form

$$\frac{T_0 - T_0^{(\mathrm{ef})}(N)}{T_0} \sim \frac{\Lambda}{\sqrt{\log N}}\,, \tag{S18}$$

with Λ a small amplitude. Similar results were obtained for other mass ratios μ and at the cold boundary. This demonstrates that the effective boundary temperatures the bulk fluid feels (induced by the boundary layers) approach the wall temperatures as N increases at a extremely slow, $\sim 1/\sqrt{\log N}$ rate, and this explains the persistent deviations found in the effective anomaly exponent in literature. More in detail, as explained above standard methods lead to an effective heat conductivity $\tilde{\kappa} \approx JL/\Delta T \sim N^{\tilde{\gamma}}$ for large enough N. Using now Eq. (S18) describing the slow decay of boundary finite-size corrections, we have that $T_{0,L}^{(\mathrm{ef})}(N) \sim T_{0,L}(1-\Lambda/\sqrt{\log N})$, so that

$$\Delta T \sim \frac{\Delta T^{(\mathrm{ef})}}{1 - \dfrac{\Lambda}{\sqrt{\log N}}}\,,$$

with $\Delta T^{(\mathrm{ef})} \equiv T_0^{(\mathrm{ef})} - T_L^{(\mathrm{ef})}$. In this way, for the effective heat conductivity

$$\tilde{\kappa} \approx \frac{JL}{\Delta T} \sim N^{\tilde{\gamma}} \sim \frac{JL}{\Delta T^{(\mathrm{ef})}}\left(1 - \frac{\Lambda}{\sqrt{\log N}}\right)\,.$$

Noting now that $\Delta T^{(\mathrm{ef})}$ is the real temperature gradient felt by the bulk fluid, we thus expect $JL/\Delta T^{(\mathrm{ef})} \sim N^{\gamma}$, so inserting this in the previous equation and taking logarithms we arrive for large N at

$$\tilde{\gamma}(N) = \gamma + \frac{\log\left(1 - \frac{\Lambda}{\sqrt{\log N}}\right)}{\log N}\,,$$

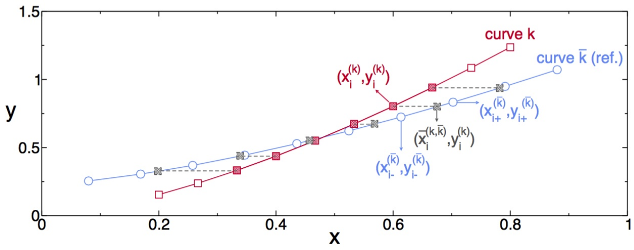

FIG. S6. **A metric to quantify data collapse.** Sketch explaining the metric used to quantify data collapse, see Eq. (S19). This metric estimates the distance between a curve k (□) and the reference curve $\bar{k}$ (◯) by measuring the average distance between each point in k and the interpolated point in $\bar{k}$ with the same y-coordinate (gray, shaded squares). Note that we restrict to points in k overlapping with the reference curve $\bar{k}$, see filled squares. The distance corresponds in this example to the average length of the dashed segments.

i.e. an effective anomaly exponent $\tilde{\gamma}(N)$ which converges at a exceedingly slow rate toward the correct, asymptotic anomaly exponent γ, in a way that closely resembles actual measurements, see e.g. Ref. [3]. This confirms that the slowly-decaying boundary finite-size corrections associated to the boundary layers are responsible of the strong, persistent finite-size effects affecting the effective anomaly exponent measured with the standard linear response method. Moreover, as our scaling method is independent of the boundary temperatures driving the system out of equilibrium, this explains why our results for the new anomaly exponent α, that we conjecture is equal to the true asymptotic exponent γ, are free of these persistent finite-size corrections.

III. A METRIC TO QUANTIFY DATA COLLAPSE

In this section we briefly explain the standard metric used in this work to quantify data collapse. This metric is based on the collapse distance first proposed in Ref. [4] and widely used in physics literature, in particular in order to obtain scaling exponents via a distance minimization procedure.

We hence consider a set of K curves, each one containing M points, and we denote this set as $\{\{(x_i^{(k)}, y_i^{(k)}), i \in [1,M]\}, k \in [1,K]\}$. The idea is now to choose an arbitrary curve $\bar{k} \in [1,K]$ as reference curve, and proceed to measure the distance of all other curves $k \neq \bar{k}$ to this reference curve along the x-direction. For that we measure the distance between each point in k and the interpolated point in $\bar{k}$ with the same y-coordinate. In order to do so, we have to restrict to points in k overlapping with the reference curve $\bar{k}$. Note also that we choose to measure distances only along the x-direction because the scaling approach developed in this paper only affects the x-coordinates of the measured curves, see Section I and Figs. 2 and 3 (right panel) in the main text. Moreover, since the chosen reference curve $\bar{k}$ is completely arbitrary, we repeat this procedure for all curves as reference curve, and average the resulting distances. In this way, our collapse metric is defined as [4]

$$D \equiv \frac{1}{\ell_{\max}\mathcal{N}_{\text{overl}}} \sum_{\bar{k}=1}^{K} \sum_{\substack{k=1\\k\neq\bar{k}}}^{K} \sum_{\substack{i=1\\i \text{ overlap } \bar{k}}}^{M} \left| x_i^{(k)} - \bar{x}_i^{(k,\bar{k})} \right| , \quad \text{(S19)}$$

where $\bar{x}_i^{(k,\bar{k})}$ is the (interpolated) x-coordinate of a point in curve $\bar{k}$ with y-coordinate equal to $y_i^{(k)}$, i.e. the projection of point $(x_i^{(k)}, y_i^{(k)})$ of curve k on curve $\bar{k}$ along the x-axis. The innermost sum over i in Eq. (S19) is restricted to points in curve k which overlap with curve $\bar{k}$ along the y-direction, i.e. those points in k whose y-coordinate is between the minimum and maximum y-coordinate of curve $\bar{k}$. In order to obtain now the projection $\bar{x}_i^{(k,\bar{k})}$ in Eq. (S19) any interpolation scheme can be used, though for our purposes the simplest linear interpolation works well. In particular, we choose

$$\bar{x}_i^{(k,\bar{k})} = \frac{y_i^{(k)} - B_i^{(k,\bar{k})}}{A_i^{(k,\bar{k})}} , \quad \text{(S20)}$$

with $A_i^{(k,\bar{k})}$ and $B_i^{(k,\bar{k})}$ the slope and the y-intercept of the interpolating function,

$$A_i^{(k,\bar{k})} = \frac{y_{i+}^{(\bar{k})} - y_{i-}^{(\bar{k})}}{x_{i+}^{(\bar{k})} - x_{i-}^{(\bar{k})}} ,$$

$$B_i^{(k,\bar{k})} = \frac{y_{i+}^{(\bar{k})} x_{i-}^{(\bar{k})} - y_{i-}^{(\bar{k})} x_{i+}^{(\bar{k})}}{x_{i+}^{(\bar{k})} - x_{i-}^{(\bar{k})}} .$$

The points $(x_{i\pm}^{(\bar{k})}, y_{i\pm}^{(\bar{k})})$ correspond to the points in the $\bar{k}$-curve bracketing point i of k-curve along the y-direction, see sketch in Fig. S6. To normalize the distance metric, we divide the resulting sums by the total number of overlapping points, $\mathcal{N}_{\text{overl}}$. Moreover, because the $L^{-\alpha}$ scaling in the x-coordinate of the measured density and temperature profiles may affect strongly the total span of the collapsed curves depending on the anomaly exponent α used, the collapse metric is also normalized by the total span in the x-direction of the curve cloud, $\ell_{\max} \equiv (x_{\max} - x_{\min})$ with $x_{\max} = \max_{k,i}[\{x_i^{(k)}\}, i \in [1,M], k \in [1,K]]$ and $x_{\min} = \min_{k,i}[\{x_i^{(k)}\}, i \in [1,M], k \in [1,K]]$, i.e. our distance is relative to the total span of the curve cloud in the x-direction.

In order to obtain the exponent α characterizing anomalous Fourier's law in our 1d fluid, we minimize the metric (S19) for varying mass ratios μ. In fact, the collapse metric $D(\alpha,\mu)$ exhibits a deep and narrow minimum as a function of α for each μ, see inset to Fig. 3 in the main text, offering a precise measurement of the anomaly exponent. Moreover, an estimate of the exponent error can be obtained from the width and depth of this minimum [4]. By expanding $\ln D(\alpha,\mu)$ around the

minimum at $\alpha = \alpha_0$, the width can be estimated as [4]

$$\Delta\alpha = \frac{\epsilon\alpha_0}{\sqrt{2\ln\left[\frac{D(\alpha_0 \pm \epsilon\alpha_0, \mu)}{D(\alpha_0, \mu)}\right]}}, \qquad \text{(S21)}$$

for a given level ϵ. Here we choose $\epsilon = 0.01$, so the estimate for the anomaly exponent is $\alpha_0 \pm \Delta\alpha$ with an errorbar reflecting the width of the minimum at the 1% level [4].

[1] del Pozo, J. J., Garrido, P. L. & Hurtado, P. I. Probing local equilibrium in nonequilibrium fluids. *Phys. Rev. E* **92**, 022117 (2015). URL http://journals.aps.org/pre/abstract/10.1103/PhysRevE.92.022117.

[2] del Pozo, J. J., Garrido, P. L. & Hurtado, P. I. Scaling laws and bulk-boundary decoupling in heat flow. *Phys. Rev. E* **91**, 032116 (2015). URL http://journals.aps.org/pre/abstract/10.1103/PhysRevE.91.032116.

[3] Chen, S., Wang, J., Casati, G. & Benenti, G. Nonintegrability and the Fourier heat conduction law. *Physical Review E* **90**, 032134 (2014). URL http://journals.aps.org/pre/abstract/10.1103/PhysRevE.90.032134.

[4] Bhattacharjee, S. M. & Seno, F. A measure of data collapse for scaling. *J. Phys. A* **34**, 6375 (2001). URL http://iopscience.iop.org/article/10.1088/0305-4470/34/33/302/meta.
